# Evaluation of synthetic training data for 3D intraoral reconstruction of cleft patients from single images

**DOI:** 10.1007/s11548-025-03396-z

**Published:** 2025-05-24

**Authors:** Lasse Lingens, Yoriko Lill, Prasad Nalabothu, Benito K. Benitez, Andreas A. Mueller, Markus Gross, Barbara Solenthaler

**Affiliations:** 1 Department of Computer Science, ETH Zurich, Zurich, Switzerland; 2 Department of Oral and Craniomaxillofacial Surgery, University Hospital Basel and University Children’s Hospital Basel, Basel, Switzerland

**Keywords:** Computer vision, Medical image processing, Registration and image fusion, Data annotation

## Abstract

**Purpose:**

This study investigates the effectiveness of synthetic training data in predicting 2D landmarks for 3D intraoral reconstruction in cleft lip and palate patients. We take inspiration from existing landmark prediction and 3D reconstruction techniques for faces and demonstrate their potential in medical applications.

**Methods:**

We generated both real and synthetic datasets from intraoral scans and videos. A convolutional neural network was trained using a negative-Gaussian log-likelihood loss function to predict 2D landmarks and their corresponding confidence scores. The predicted landmarks were then used to fit a statistical shape model to generate 3D reconstructions from individual images. We analyzed the model’s performance on real patient data and explored the dataset size required to overcome the domain gap between synthetic and real images.

**Results:**

Our approach generates satisfying results on synthetic data and shows promise when tested on real data. The method achieves rapid 3D reconstruction from single images and can therefore provide significant value in day-to-day medical work.

**Conclusion:**

Our results demonstrate that synthetic training data are viable for training models to predict 2D landmarks and reconstruct 3D meshes in patients with cleft lip and palate. This approach offers an accessible, low-cost alternative to traditional methods, using smartphone technology for noninvasive, rapid, and accurate 3D reconstructions in clinical settings.

**Supplementary Information:**

The online version contains supplementary material available at 10.1007/s11548-025-03396-z.

## Introduction

The cleft lip and palate is the most common craniofacial birth defect with an estimated prevalence of 1 in 700 [1]. In the last decades, the presurgical orthopedic (PSO) treatment is commonly used to narrow the cleft and reduce the number of surgeries on an individual patient [2]. The main step of the treatment is designing a plate that fits the patients cleft and palate precisely. This plate provides a narrowing of the cleft by prohibiting the tongue from entering the cleft. In addition, this plate supports early speech development [3, 4] and a more natural food consumption. To create such a plate, the practitioner first needs to acquire a patient-specific intraoral model. Next, the practitioner designs a plate according to the model, such that it fits accurately into the patients intraoral area.

The design of such a plate can nowadays be automated in a digital environment that reduces time cost of the healthcare professional during the creation process [5]. To create a 3D model of the intraoral region, 3D scanners are deployed to capture and digitize the model. In comparison, the capture through manual impression requires additional security measures as well as specialized healthcare workers on standby during the process [6, 7]. While the 3D scanner carries less risk, it has higher acquisition cost, requires additional training and is often tied to third-party Software Licenses without which the hardware does not provide the same results or does not function. These requirements make the 3D scanner less accessible in low and middle-income countries. Therefore, our goal is to provide a safe and easy to use method, that is low cost and generates the model of the intraoral region of a patient with cleft lip and palate.

We take our inspiration from the work of single-image 3D face reconstruction of Wood et al. [8]. These methods usually rely on two main steps. First, they detect a set of well defined landmarks on an image. These landmarks correspond to specific, anatomy-defined features, such as the corner of the eye or the tip of the nose. Next, they fit a shape model to the predicted landmarks. The shape model is a problem-specific prior, in this case a face prior, that can be fit by minimizing a cost function of the 2D landmarks and 3D points. One drawback of this approach is that a large amount of data is needed, both, annotated 2D images and 3D meshes. While there are public face datasets and face models that fulfill these requirements, such as Bansal’s et al. [9] dataset, they do not exist for images or meshes of patients with cleft lip and palate. However, there are also results in the face reconstruction field that show the possibility of synthetic training data and the transfer to in-the-wild images, such as Wood et al. [10]. The advantages of synthetic data are significant. For one, it allows for perfect annotation of 2D landmarks. In addition, synthetic data can sometimes be artificially created to expand the existing dataset, create more variety during training, and allow for better generalization of the trained model.

**Fig. 1:**
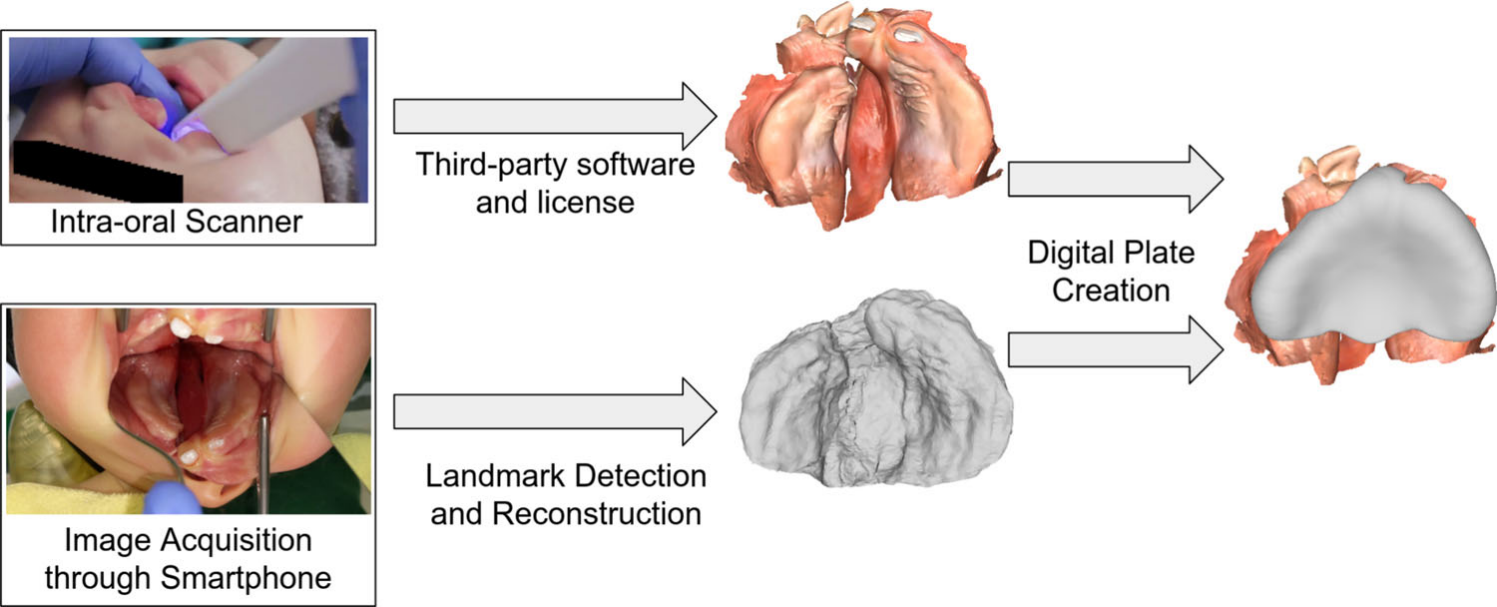
Image shows an overview of the current capture process and plate creation for PSO treatment in our partner hospital (top row) and the capture process using our method (bottom row). The clinical method requires expensive intraoral scanners and proprietary software. Our method uses a smartphone and our code, published with this paper. After reconstructing a 3D mesh, the automated digital plate design [5] can create an orthodontic plate for treatment in both cases

We therefore aim to show the applicability of synthetic training data to predict landmarks on cleft images and reconstruct the cleft mesh with a shape model. We further evaluate the potential of a larger dataset size to bridge the domain gap between synthetic and real images. Some of the advantages of this approach are low-cost hardware, fast reconstruction, and independence from licensed software. The only necessary tools to capture the palate area are a mirror and a smartphone. Our approach requires a single image compared to the photogrammetry approach of Lingens et al. [11], which required a static scene. Only video captures of anesthetized infants are still enough and lead to a successful reconstruction. It would further enable remote reconstruction, which, in combination with digital plate creation, would allow for full remote capture, shape reconstruction, plate creation and plate printing. We compare the current medical practice of our partner hospital with our approach in Fig. 1.

## Related work

Synthetically trained networks for single-image reconstructions on faces have shown consistently promising results and sparked our curiosity toward the applicability of similar methods in a medical environment, namely the cleft reconstruction. We focus our following discussion on single-image-reconstruction, data-driven shape models and synthetic training data.

***Single-image reconstruction:*** Single-image reconstruction focuses on the retrieval of 3D geometry from a single monocular image. Some methods focus on the initial estimation of a depth map, such as Saxena et al. [12] and reconstruct a 3D surface with the depth information. Other approaches focus on initially finding specific points and regions of interest and then fitting a 3D shape model to those detected objects. Wu et al. [13] detect edges and contours of teeth on a single image with image processing methods and fit a teeth shape model to the observation. Multiple approaches for faces, for example Wood et al. [8], initially detect the face in an image and crop to that region. Next, they predict 2D landmarks on the image with a pre-trained neural network. Finally, they fit a principal component analysis (PCA) face model to the detected landmarks, often optimizing for identity, expression and pose.

***Data-driven shape models:*** Data-driven shape models are originally founded on the basis of PCA. After acquiring a multitude of shapes (3D meshes), one registers them to the same template model to create correspondence between every point of the mesh. The lower eigenmodes are often removed, as they represent only a small part of the variance. This leads to a compressed model that creates reasonable results within the initially observed distribution of shapes. A large quantity of shape model applications focuses on face reconstruction [14, 15]. Recent methods explore the performance of transformers for faces from monocular images [16]. They utilize 2D and 3D data for learning and are able to disentangle different facial components while they keep temporal stability. With the rise of learning-based methods, new neural shape models have been designed. One such neural shape model is DeepSDF [17]. There are other learning-based methods that build on a similar concept but further introduce hierarchy structures to improve high-frequency details [18, 19]. However, PCA-based models still remain widely used in many fields and are our method of choice due to their comprehensibility and ability to produce reasonable in-distribution results.

***Synthetic training data*** Synthetic data offers many benefits over its real counterpart but comes with drawbacks as well. With the dominance of learning-based methods in most computer science fields, results of a given training experiment heavily depend on the amount and quality of the accessible data. Especially in the medical field, the acquisition of data is difficult. Patient confidentiality contributes a large amount to this challenge and can prohibit publication of collected data, further reducing accessibility. In addition, the non-uniform processes of hospitals to capture and store the data pose more challenges when collecting data. Synthetic training data is, therefore, an alternative to consider, especially as the feasibility to bridge the domain gap from synthetic training into real-world prediction has been proven [10]. Recently, Hewitt et al. [20] tackled the challenging task of capturing the entire human body pose, as well as facial expression and hand tracking. Their work relies solely on synthetic training data but is applied to real-world footage and, therefore, tackles the domain gap. Not only can synthetic data be synthesized to create more samples, it also provides increased control over the precision of training data. Real-world data often requires manual annotation and includes inaccuracies or even errors. Thus, we focus on synthetic training data, to leverage the much larger dataset size and reduce annotation errors.

To our knowledge, the only other work focusing on cleft reconstruction from smartphone capture is Lingens’ et al. [11] work. They focus specifically on cleft reconstruction from multiple RGB-images and rely on photogrammetry. This process takes multiple hours and requires a relatively static environment. To achieve a static scene, infants are usually anesthetized during the capture process. We, therefore, focus on a solution that works in seconds and on a single image, resulting in a less sensitive capture process. This improves the experience for both patients and healthcare professionals.

## Methods

We present a solution that works on single images and reconstructs a 3D model. Our method is heavily inspired by Wood et al. [8]. We first create two datasets, a synthetic and a real one. Then, we train and validate our landmark prediction on the synthetic dataset and test on the real dataset. We further utilize a shape model that is fit to the landmarks and reconstructs the final 3D mesh.

### Dataset

A dataset consists of images and corresponding 2D positions per image representing the landmarks. We discuss the creation of each dataset in the following subsections. Each identity in our dataset can have multiple meshes. In this case, meshes are acquired from the same patient but at different ages.

**Fig. 2:**
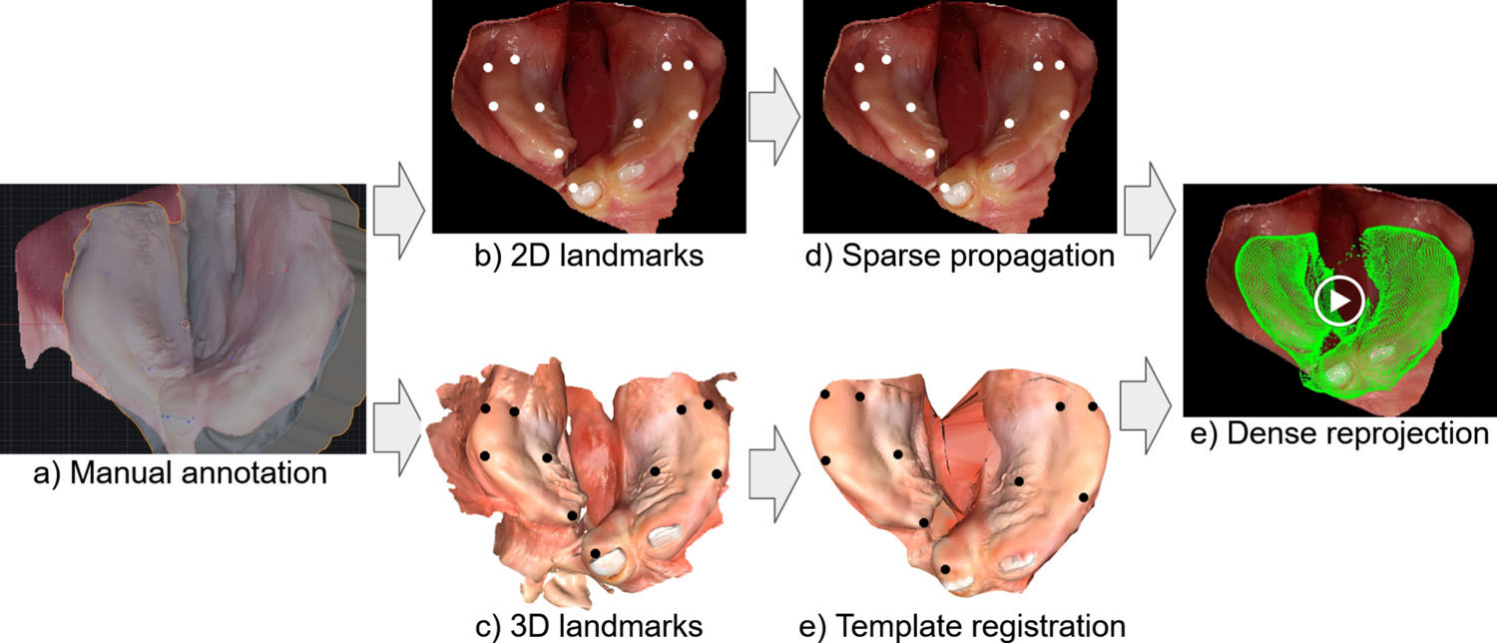
We present our method to create dense annotation on real images of videos. We manually annotate paired images and meshes of each identity (**a**). We export each information individually (**b**, **c**). Next we utilize the sparse annotated 2D landmarks (**b**) together with pixel tracking to propagate the 2D annotations through the entire video (**d**). Further, we register our template mesh to the original scan and transfer the landmarks (**e**). Finally, we reproject the 3D landmarks into the image space of each individual frame and create dense reprojection for the entire video (**e**)

#### Real-world data

The real-world dataset consists of pairs of real images, in our cases frames from multiple videos, and their corresponding 2D points. This real-world dataset relies on pairs of respectively a mesh and a video of the same patient at the same time. An overview of the process is visualized in Fig. 2. To create such 2D-to-3D correspondences, we first create sparse correspondences between the mesh and a single image of a video. We use our custom designed blender [21] tool to annotate both 2D and 3D points. First, the user chooses a point on the 3D mesh and then drops the corresponding point on the 2D image. The tool allows for a transparent overlay of the mesh to ensure precision in landmark placement and automatically aligns the mesh to the image from a given viewpoint after at least four chosen correspondences. Each consequent correspondence further optimizes the alignment, iteratively refining translation, rotation and scale to minimize the least squares error between the 2D landmarks and 3D points projected into 2D space. The following formula expresses the projection of 3D points into 2D images. **K** is the camera intrinsic matrix, consisting of the focal length ($f_x$,$f_y$) and the principal point $(c_x, c_y)$. $\mathbf {[R|T]}$ is the combined rotation and translation matrix, and *s* is the scalar scale. The coordinates (*u,v*) are represented in image space and $(X_w, Y_w, Z_w, 1)$ are the homogeneous world coordinates of the corresponding 3D points:

1 $$\begin{aligned} s \begin{bmatrix} u \\ v \\ 1 \end{bmatrix}&= \textbf{K} \hspace{0.1em} \mathbf {[R|T]} \begin{bmatrix} X_{w} \\ Y_{w} \\ Z_{w} \\ 1 \end{bmatrix}\nonumber \\  &= \begin{bmatrix} f_x &  0 &  c_x \\ 0 &  f_y &  c_y \\ 0 &  0 &  1 \end{bmatrix} \begin{bmatrix} r_{11} &  r_{12} &  r_{13} &  t_x \\ r_{21} &  r_{22} &  r_{23} &  t_y \\ r_{31} &  r_{32} &  r_{33} &  t_z \end{bmatrix} \begin{bmatrix} X_{w} \\ Y_{w} \\ Z_{w} \\ 1 \end{bmatrix} \end{aligned}$$

We approximate the intrinsic matrix and optimize for the rotation angles, the translation vector and the scale.

We annotate one frame per video this way, with around 12 correspondences each. Afterward, we use long-term tracking of pixels [22] to propagate the 2D landmarks through the entire video. Next, we register a template mesh to the original mesh with non-rigid iterative closest point to create dense 3D correspondences between all meshes. We then transfer the annotated 3D points on the original mesh onto the template mesh. We project the template mesh into all frames of a video, using the transferred 3D points and their propagated 2D correspondences. This creates a set of dense annotated landmarks on each image. In our case, the template mesh consists of 10.000 vertices and, therefore, each image is paired with that many annotations. Note that the amount of points is solely dependent on the resolution of the template mesh and can be adapted as desired, without additional manual annotation work. The manual annotation, the propagation and the reprojection can all introduce errors in the positioning of the final landmarks. Therefore, we filter out images based on a quality assessment, consisting of the number of points that are within image boundaries, the reprojection error, and the rejection of large changes in the reprojection matrix between neighboring frames. Finally, our dataset consists of 13.014 images of 21 videos from 12 different identities.

#### Synthetic data

**Fig. 3:**
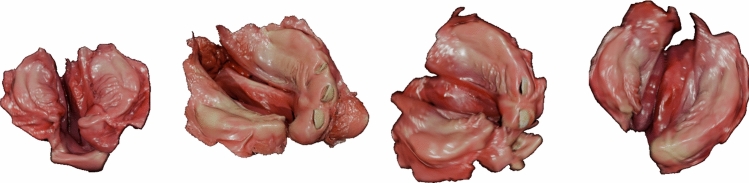
An array of synthetically renders samples of different identities

The synthetic dataset consists of pairs of artificially rendered images and their corresponding 2D landmarks. These images are generated using textured 3D intraoral scans of a patient, which provide realistic color information essential for accurate texture representation. The dataset is created by loading both the original 3D scanned mesh and the registered mesh into Blender software [21]. Collaborating with an artist, we defined realistic material properties, appearance parameters, and rendering settings to closely match the visual characteristics observed in real videos, with the color information directly provided by the intraoral scans. To generate varied and realistic images, we apply augmentations such as randomized camera positions, angles, and motion blur. Unlike approaches that rely on shape models to generate artificial shapes and textures for a large number of synthetic data, our approach uses real, textured patient scans, which are limited in availability. However, this trade-off ensures high-fidelity texture representation, which is critical for realism. For each rendered image, we disregard the template mesh but record the vertex positions in image space as landmarks, ensuring precise landmark placement. The final dataset comprises 100 images per mesh, derived from 90 meshes representing 65 patients. Example images are provided in Fig. 3.

### Landmark prediction

The landmark prediction consists of a convolutional neural network (CNN) structure that predicts the 2D position of the landmarks based on a single input image. We train the network with a negative-Gaussian log-likelihood loss:

2 $$\begin{aligned} \mathcal L = \frac{1}{2} \left[ \log \ \textrm{max}(\sigma ^2,\textrm{eps}) + {\frac{(y-\mu )^{2}}{\textrm{max}(\sigma ^{2},\textrm{eps})}}\right] \end{aligned}$$

Here, *y* is the reference annotation, $\mu$ is our prediction, $\sigma$ the confidence of the prediction, and eps the machine epsilon. This loss predicts the position of a landmark as the mean of a Gaussian distribution and in addition the certainty of that prediction as the sigma of the distribution. The loss penalizes uncertainty as well as a wrong prediction with high certainty. The network is then trained on the synthetic dataset. To increase robustness and generalizability, we further apply a variety of augmentations to the images, such as random rotation and flipping, color shifts and added noise patterns. We further sub-sample the 10.000 points to 1.000 and have a higher sample rate in the plate’s contact regions on the ridges. The sub-sampling barely changes quality, but improves performance significantly. Finally, we train until convergence and verify the results on the real-image dataset. The inference time is  100ms per image. Additional details of our configuration is listed in the supplemented material.

### PCA model fitting

The PCA model fitting takes as input 2D landmark positions corresponding to the points of the template shape. We do an iterative flip-flop approach. Compared to facial fitting such as [8], we do not optimize for joint positions or expression and only optimize for the shape model and its 3D transform. We, therefore, optimize alternating between rotation, translation, and scale and the weights of the eigenvectors. Each optimization is fitted until convergence. Once both optimizations have converged in the same iteration, the fitting terminates. During the fitting, any subset of points can be chosen to emphasize specific regions, such as the ridges of the palate, with which the treatment plate is in contact. The fitting takes around 40 s per image for 1.000 points. However, this can be sped up by reducing the number of points, improving the initial guess and adapting the learning rate.

## Experiments and results

We present the results of our method and evaluate its performance on real-world data, focusing on unilateral clefts due to the availability of the largest number of samples, intraoral scans, and videos for this cleft type. To understand the scalability of our approach, we conduct experiments with datasets containing varying numbers of identities (patients). By varying the dataset size, we explore how the performance of our pipeline changes as more data becomes available. This evaluation provides insights into the potential for improvement as the dataset grows, and more intraoral scans from diverse patients are collected. Experiment details are in the supplements.

### Concept verification

**Fig. 4:**
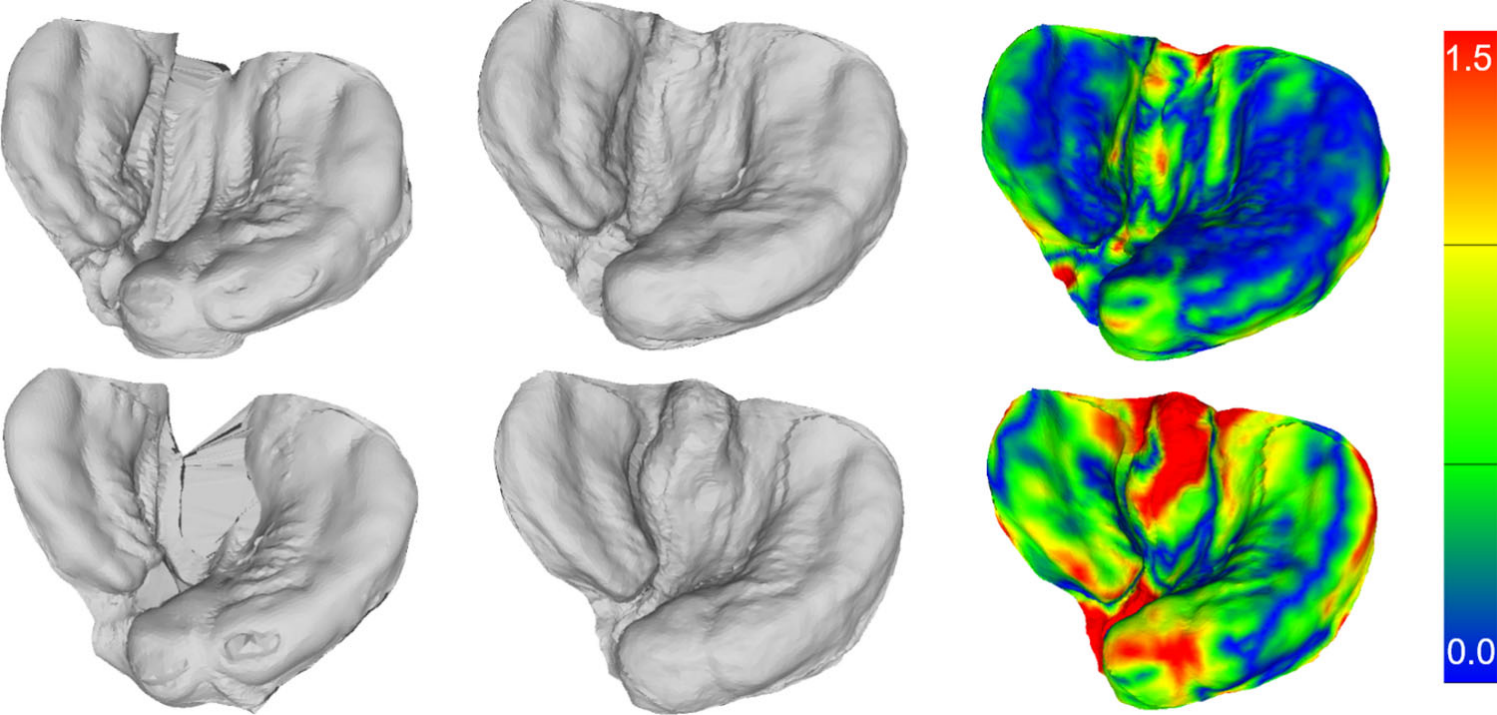
We fit the PCA model to 2D landmarks and present the results. Each row represents a different identity. The columns show from left to right: the registered template to the identity, the PCA reconstruction to the identity, the color-coded error of PCA reconstruction

We first verify our method on cleft images by reconstructing the shape model to perfectly reprojected points. This experiment measures the error created by dropping the third dimension when projecting the points into image space. We show the results in Fig. 4. The resulting 3D mesh can serve as input for the digitized plate creation process [5] as its quality is sufficient. For the reconstruction quality to be considered clinically precise enough, the plate contact area should have an average error below 0.5 mms. Further details on the clinically required precision can be found in the supplemental material.

**Fig. 5:**
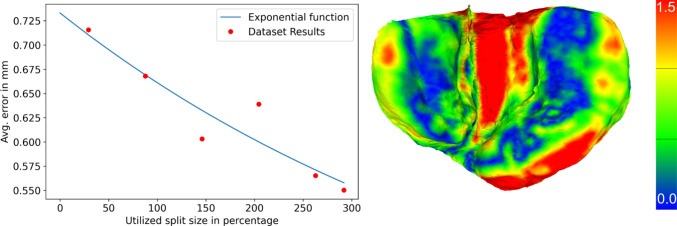
We demonstrate the reconstruction error of the PCA model dependent on the amount of different meshes used to create the model. We set the number of eigenmodes such that they capture 99.9% variance, respectively, for each model size. We fit an exponential function to our data points. We visualize an example reconstruction of the PCA model with color-coded error compared to the registered mesh. Note that the contact regions on the ridges, which are highly relevant, have usually a lower error than the less relevant non-contact regions on the inside and the outer edges

Additionally, the quality of any shape model reconstruction depends on the quality of the shape model itself. Shape models can consist of multiple thousands of identities [15]. We utilize 292 different meshes of 224 different identities. In Fig. 5, we show the model reconstruction quality dependent on the number of samples. The convergence point estimated by the fitted function is 0.340 mm. The current optimum of 0.550 mm is far higher than the convergence point and suggests significant improvements with more data.

Next, we test the theoretical ceiling of the network’s landmark predictions. To maximize the prediction quality, we train the network on multiple rendered images of a single identity. While the validation set remains unseen during training, the images have a very high correlation. The results of this experiment indicate that the network can create precise enough predictions for our medical target and its predictions are almost indistinguishable from the training data. While this is expected when training on the same identity, it validates the approach.

**Fig. 6:**
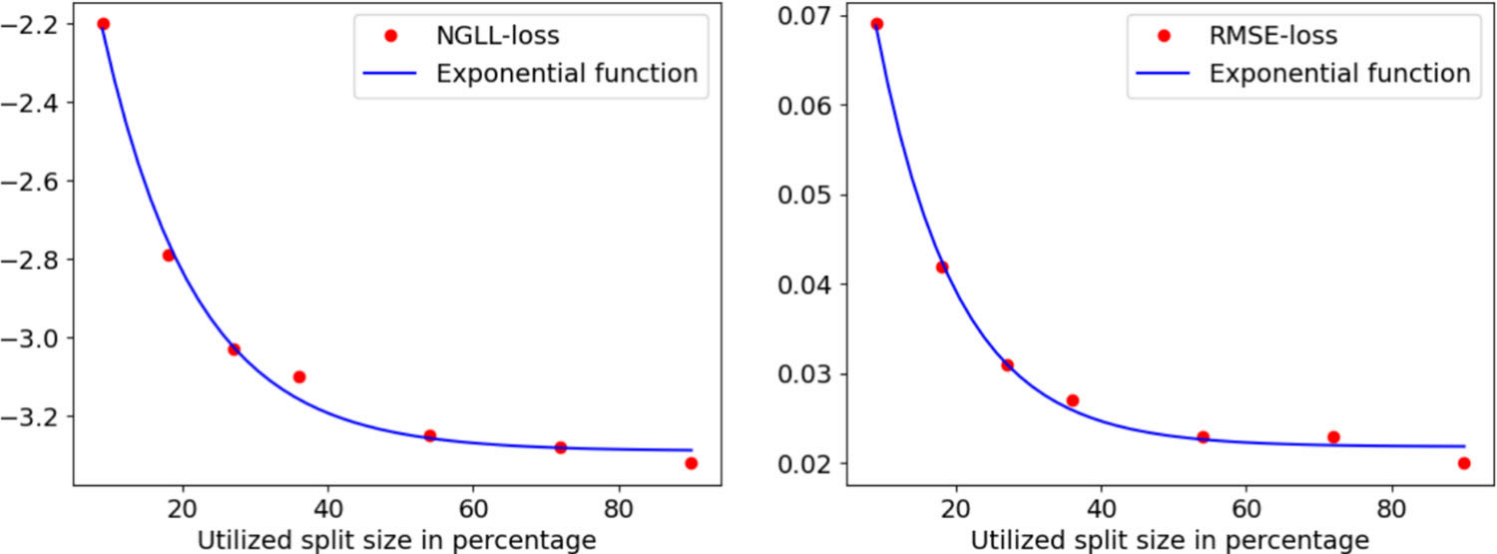
We show the validation error on synthetic data and fit an exponential function to it. On the left, we show the negative-Gaussian log-likelihood loss. On the right, the root-mean-square error loss. In both cases, it is evident that an increase in the number of identities in the training set improves the performance. However, the performance on the synthetic dataset is estimated close to the convergence point

**Fig. 7:**
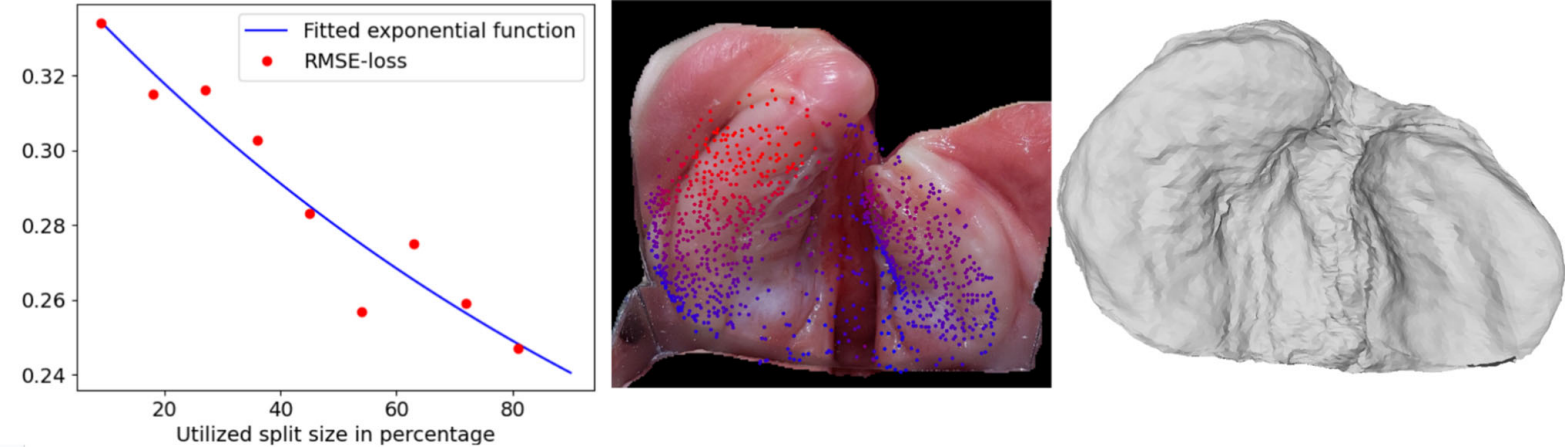
We show the performance of the synthetically trained dataset on real images. On the left, we show the quantitative performance, depending on the number of different identities in the training set. In the center, we show a prediction on a real image, and red indicates a larger distance to the real landmarks. On the right, we see a reconstruction of our shape model based on the prediction

### Synthetic training and verification

We want to evaluate the performance of our method on both synthetic and real test data. First, we evaluate on the synthetic dataset and present the results in Fig. 6. On synthetic data, the results are promising. The quality of the highest sample size is close to the quality necessary for the PSO, depending on the specific identity it can be either exceeding the requirements or not quite fulfill them. We can see that both the 3D error and the landmark prediction error are converging with increasing sample size. We can further see that the convergence point is not yet reached, indicating further possible improvements with increased sample size. The same results can be observed in Fig. 7, where we test the synthetically trained network on in-the-wild images. The overall error is higher than on the synthetic data. This is expected and represents the domain gap challenge. The convergence point of the RMSE loss estimated by the fitted function is 0.022 for the synthetic tests and 0.138 for the real data tests. This indicates that the performance on real data can still be drastically improved, while the performance on synthetic data is close to its optimal point.

## Conclusion

We present a single-image cleft reconstruction method. The method is inspired by the field of single-image face reconstruction, which has proven the applicability to real-world images and the possibility to train on synthetic data to achieve in-the-wild results. We create a synthetic and real-world dataset of annotated cleft images to evaluate the method for cleft reconstruction. We present experiments that verify the feasibility of this approach for clefts and analyze its performance on our dataset. We conclude that given the current dataset size, the performance for real-world images is not sufficient. However, acquiring a larger dataset promises great improvements of both, the prediction model, and the PCA model reconstruction. The success of single-image cleft reconstruction will be mostly contingent on the realistic expectation of collecting more intraoral cleft scans.

## Limitations and future outlook

While the current synthetic dataset is limited in size, as it relies on augmented intraoral scans from real patients, we view this as an opportunity for future growth. As more real patient data become available, we will be able to refine and validate our approach on these images, bringing us closer to clinical applicability. The promising results from our synthetic dataset, along with the evaluation and extrapolation, provide a strong foundation for continued progress.

Looking ahead, there is significant potential for further expansion of the dataset. For instance, incorporating a texture model could enable the inclusion of digitized impressions, which, while derived from real patients and providing accurate anatomical shapes, lack texture information due to the absence of intraoral scans. Additionally, combining an expressive shape model with a texture model could generate new, realistic shapes and textures, further increasing dataset size. However, achieving this requires large numbers of intraoral scans to develop shape models that capture the fine details of patient anatomy, and synthesizing realistic textures for these models remains a significant challenge. These considerations underscore the importance of balancing dataset expansion with the need for high-fidelity representations. In the future, we hope sufficient data samples will be collected to further validate our evaluations, ultimately making cleft reconstruction more accessible by reducing costs and eliminating reliance on third-party software or time-intensive professional training. Beyond cleft reconstruction, we believe our approach holds promise for other areas of medical applications. Just as we successfully adapted face reconstruction techniques to cleft reconstruction, similar methodologies could be applied to other fields, provided there is access to enough 3D models. By applying our data synthesis and augmentation techniques, other areas such as dental or craniofacial surgery could benefit from improved, cost-effective solutions that rely on realistic 3D model generation, reducing the need for manual interventions and accelerating clinical workflows.

## Supplementary Information

Below is the link to the electronic supplementary material.

Supplementary file 1 (pdf 13673 KB)
