# Supplementary material for: Evaluation of synthetic training data for 3D intraoral reconstruction of cleft patients from single images
Source: Int J Comput Assist Radiol Surg. 2025 May 24;20(7):1471–9. doi: 10.1007/s11548-025-03396-z (PMC12226614; doi:10.1007/s11548-025-03396-z)
Supplement: Supplementary file 1 — (pdf 13673 KB) [file 11548_2025_3396_MOESM1_ESM.pdf]

Scan Hardware:
The 3D intraoral scans are acquired with a Medit-i500 (https://www.medit.com/medit-i500-intraoral-scanner/) and utilize the according medit software (https://www.medit.com/medit-link/)

Data Sharing and Reproducibility
Datasets: Given the ethical considerations in handling patient data, particularly the sensitivity of medical information from children and partial facial data, we cannot share our entire dataset. Both, the real and the synthetic data, are directly based on the intraoral region of patients, which can in theory be used to identify the patient. We aim to publish a small, anonymized subset of data from patients who have explicitly consented to its use for scientific purposes, demonstrating our commitment to ethical data sharing.
PCA Model: We will publish our PCA Model of the cleft shape, which is also the first published unilateral cleft shape model.
Code: We will publish our code together with instructions and parameters on how to train and validate with our method. We will further publish our trained network weights. The config file to produce our best results will be provided. We will further present guidelines to create a custom dataset and train with it. The code includes our methods to reconstruct 2D landmarks or 3D correspondences with a PCA Model. We will further publish the blender code to augment the render pipeline of an input IO mesh, to generate synthetic image data.

Experiment setup
We ran our experiments on a RTX2080 Ti. The memory footprint is at most 12GB of GPU-memory, but can be adapted by tuning the batch size, image size or the choice of the backbone.
For our training parameters we present the final value and the tested alternatives in brackets:

1.	Learning Rate:	1e-3	(1e-2, 1e-4)
2.	Batch size:	16	(4 ,8, 16, 24)
3.	Optimizer	AdamW	(Adam)
4.	Stop Patience	30	(20, 30, 50)
5.	Max No. Epochs	300	
6.	Loss	NGLL	(RMSE, RSE)
7.	Backbone	convnext_base	(resnet, densenet, vit, convnext)
8.	Image size	256	(512)

For the parameters 1-3 no or low sensitivity was detected. For 4 the stop patience in combination with batch size is relevant, as with small batch sizes a low patience leads to termination before convergence. For 5 the maximum number of epochs was never reached. For 7 we tried the listed backbones and their varied sizes. Convnext performed the best in combination with the NGLL loss. However, resnet and denset had only slightly worse results. For 8 the results between 256 and 512 performed similarly, but the training and validation speed dropped significantly by ~4x.

We further applied a variety of image augmentations during training to increase the robustness of our network.
First we applied a squared padding, without changing the aspect ratio. Next we resize the images to 256 width and height. Next we apply horizontal and vertical mirroring, each with

respectively 50% probability. Further, we rotate the image by a random angle between -30 and 30 degrees. Then, we apply pytorch's gaussian blur with a probability of 10%, a kernel of (5,9) and a sigma of (0.1,5.0). We apply the pytorch color jitter function with a probability of 30% and brightness=0.5, contrast=0.5, saturation=0.5 and hue=0.3.
Finally we normalize the landmarks, such that every landmark that is on the image (i.e. lies between 0 and 256 on the x and y axis) now lies between -1 and 1 on the x and y axis, as neural networks tend to learn better in the range.

Figure 1 shows the initial comparison of different backbones. Note that we do not normalize the landmark errors in this figure. We observe that densenet121 and both variations, resnet101 and resnet50 perform similarly. As resnet50 is the simplest and fastest model of the three models to train and infer without dropping performance significantly, we choose resnet50 as the backbone of our model.

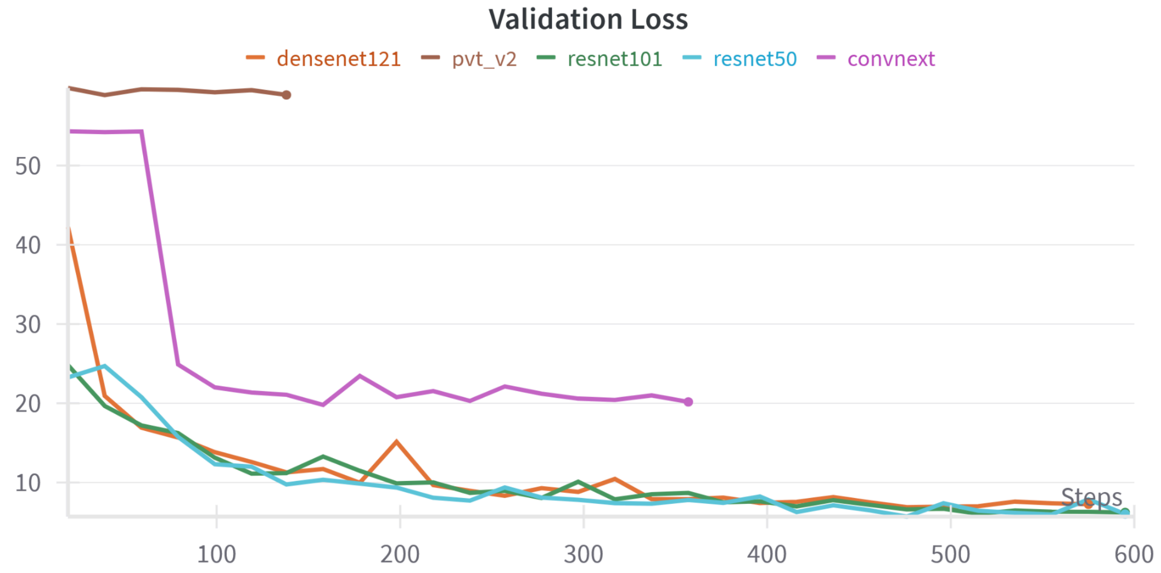

Figure 1: We show the unnormalized validation loss of different backbones when predicting landmarks.

Figure 2 shows the error scaling of the PCA reconstruction with the amount of predicted landmarks. The complexity, learning time and inference time of the model increases with the amount of landmarks used. However, the accuracy improvements are minor after ~1000 landmarks. We therefore subsample 1000 landmarks for training and fitting. We focus our subsampling more on the contact region of the plate to achieve higher accuracy in these areas.

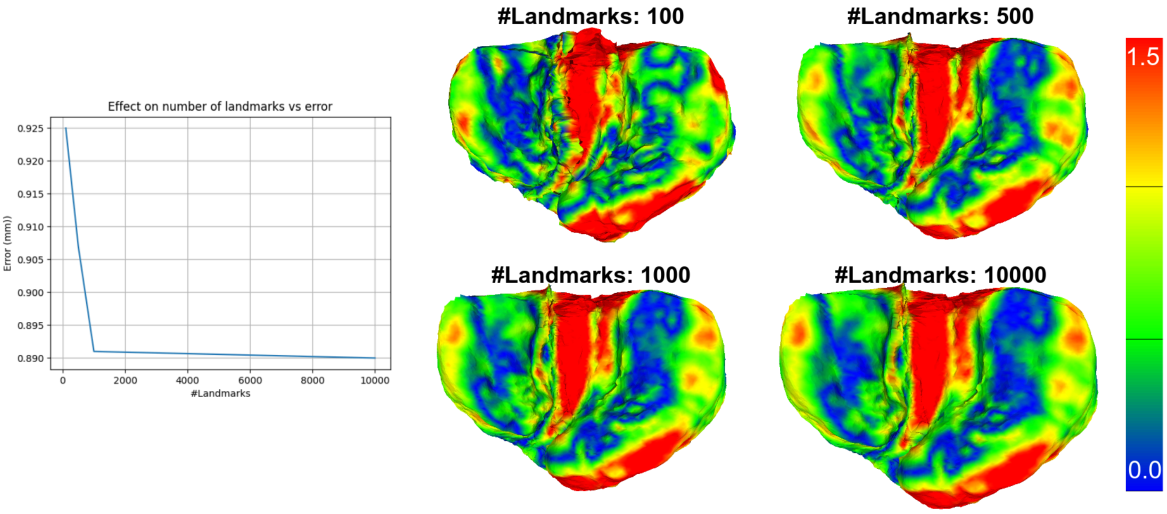

Figure 2. On the left we show the error in mm of the PCA reconstruction when fitted to reprojected landmarks. We experiment with multiple numbers of landmarks and notice a significant drop off in accuracy improvement around 1000. On the right we provide error maps of the same reconstruction when fitted to different numbers of landmarks.

Figure 3 shows the error scaling of the PCA reconstruction with the amount of used eigenmodes of the PCA model. The reconstruction time of fitting the PCA model to landmarks increases significantly with more eigenmodes. However, the accuracy improvements are minor after ~150 eigenmodes. We therefore choose 150 Eigenmodes in our reconstructions, unless we specify other values.

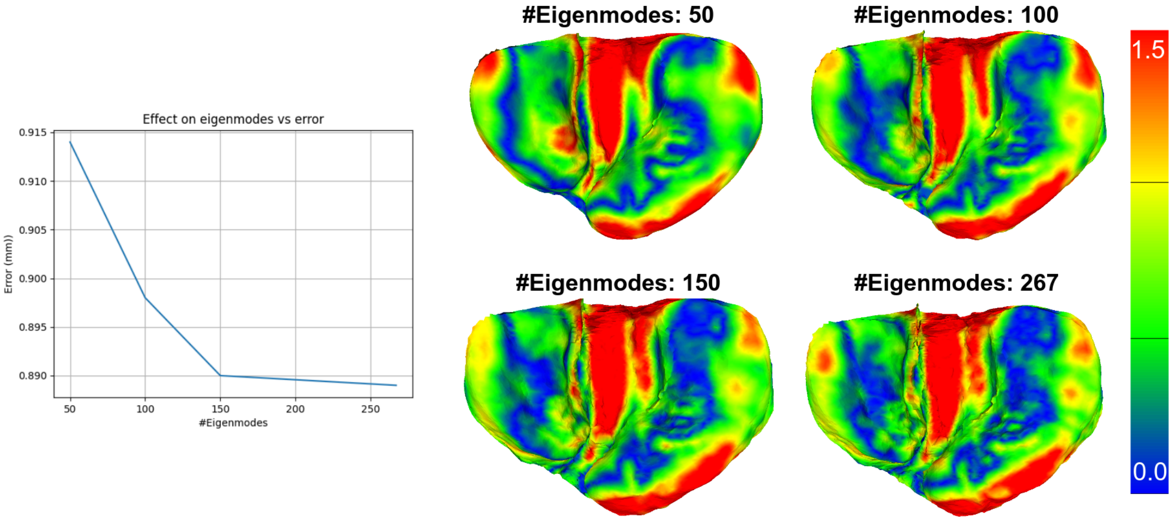

Figure 3: On the left we show the error in mm of the PCA reconstruction dependent on the eigenmodes. We notice that after 150 eigenmodes the improvements become significantly smaller. On the right we provide error maps of the same reconstruction when fitted with different amounts of eigenmodes.

Fine-tuning on mixed datasets:
We further investigate the improvements that would come with training first on a synthetic dataset and then do additional training on a mixed synthetic and real dataset. As our real dataset is small, we combine it with the synthetic to avoid overfitting. Due to the small number of identities in the real set, the validation set is too small to make robust statements about the performance. However, initial results show promising improvements compared to solely training on synthetic data.
Figure 4 shows a qualitative prediction and *Figure 5* shows the RMSE loss on the validation set. Note that loss values below 0.1 are quickly reached. This exceeds the estimated convergence point of 0.13 when relying only on synthetic training and far exceeds the current loss on real-world data of 0.25.

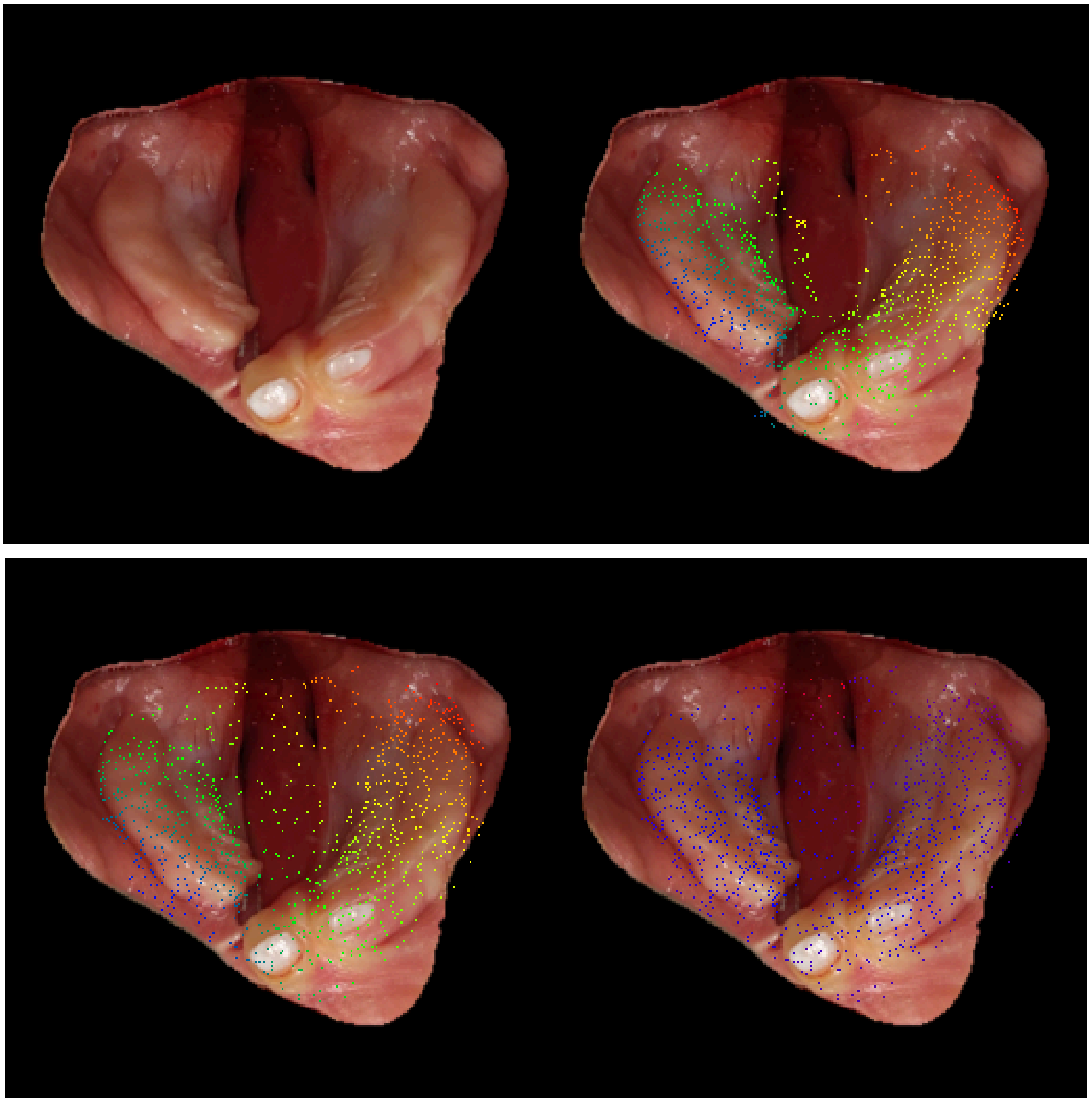

Figure 4: We show a qualitative landmark prediction on a real image after fine tuning our network on real and synthetic data. In the top row from left to right we have the original image and the ground truth reprojection. In the bottom row from left to right we have the predicted points and the confidence of the predicted points. Blue is more confident. Note that the coloring of the points in the top right and bottom left does not represent error or confidence but rather each color corresponds to the same point. This allows for visual comparison if the same indexed points are predicted at the same location.

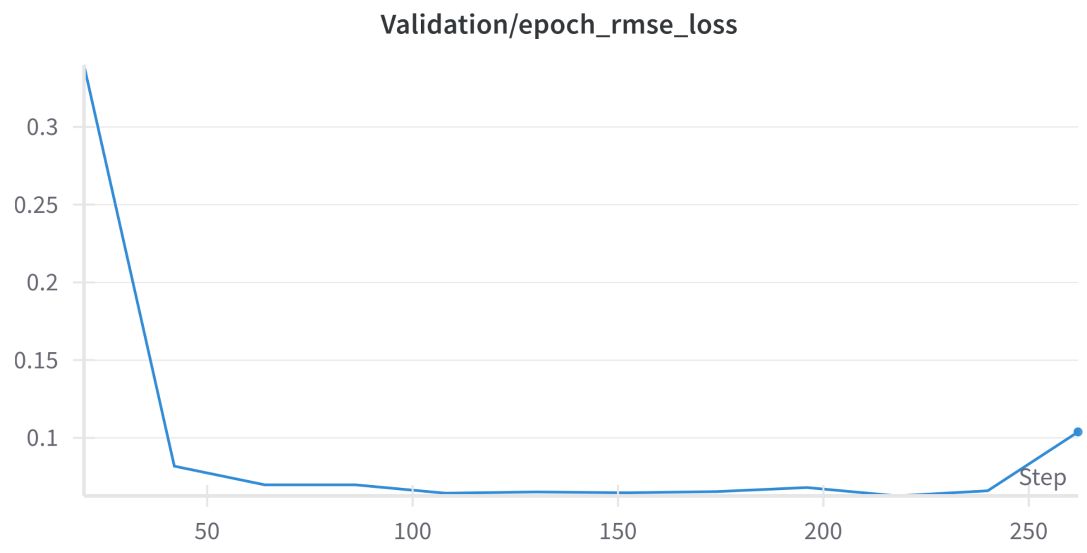

Figure 5: We show the improvement of the landmark prediction when fine-tuning on a dataset consisting of both: real and synthetic data.

Clinical Accuracy:
The scientific research on cleft reconstruction is extremely limited. There exists no global accepted standard on how to measure and validate results. In discussions and tests during our research, a heuristic arose that the contact area of the plate should be the main focus and errors should mostly not exceed 0.5mm in this region. For reference, the entire cleft mesh has a diameter of approximately 40-50mm. The error is calculated by comparing the reconstruction to the captured IO scan. For the non contact regions of the plate, the most important feature is that the shape is precise enough that there is no collision with the plate. We visualize the contact region and some plates in *Figure 6*. We further refer to the work of Schnabel et al. [2] for more in depth discussion of plate creation and the requirements for the input mesh. They discuss the evaluation and quality assessment of the resulting plate. Lingens et al. [1] offers another example of evaluating the final results for clinical applicability with the same method.

Once an approach shows results with the desired accuracy, orthodontic plates should be generated on the original scan and the reconstruction and sent to the healthcare professionals for comparison and qualitative analysis. The assessment of the doctors is the final evaluation for clinical applicability. We abstained from the plate creation and final evaluation, as our method did not reach the desired accuracy.

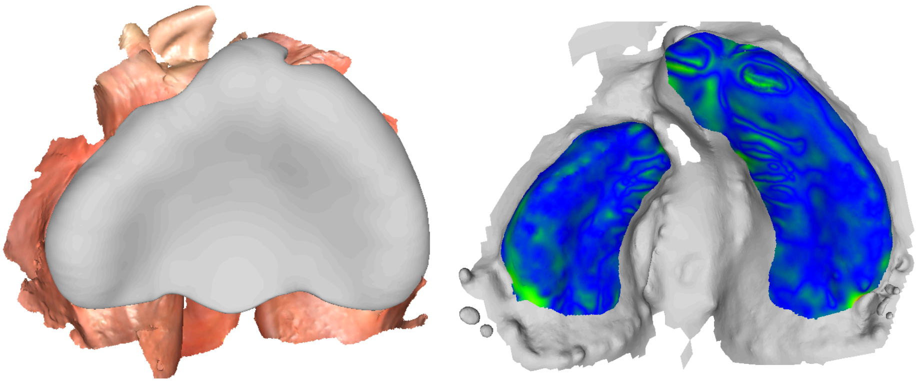

Figure 6 We visualize a plate and the contact region. The contact region is the area of interest when evaluating a cleft reconstruction, as the orthodontic connects with the intraoral area here.

Domain Gap:
The domain gap represents the difference in validation performance on synthetic data and real data. We thank reviewer 2 for the suggestion to plot both graphs in the same figure. This allows for easier estimation of the domain gap. While the convergence point of the validation on real data is higher than the performance on synthetic data, we believe that the domain gap can be narrowed further. The first indication is the above reported fine-tuning results. *Figure 7* shows the two RMSE convergence plots presented in Fig5 and Fig6 of the paper in the same plot. We fit exponential functions to estimate a convergence point that can be reached with more data. We estimate that further data samples, synthetic and real, lead to even larger improvements. Therefore, a combination of fine-tuning on real-world data, collecting more data samples and other future improvements could narrow the domain gap in future work even further.

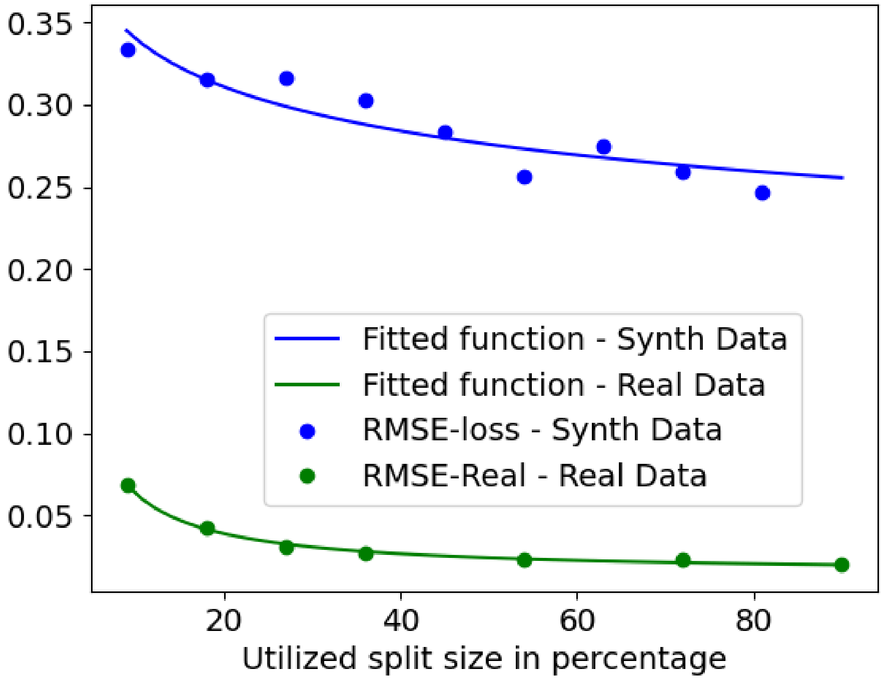

Figure 7: We plot the error of landmark prediction when evaluating on synthetic and when validating on real-word data. The direct comparison highlights the domain gap.

Qualitative Results & Comparison to previous methods:
We show qualitative results of our method in *Figure 8*, the PCA reconstructions and the corresponding error maps. We further provide the reported error of the photogrammetry approach by Lingens et al. [1] for comparison in *Figure 9*. Our current approach does not achieve the quality that the photogrammetry approach can achieve. However, our method is faster, has less requirements during capture and has less failure cases. In addition our method has tangible points of improvement. Note that the error measurements in *Figure 8* are not focused only on the region of interest when compared to the error values in *Figure 9*.

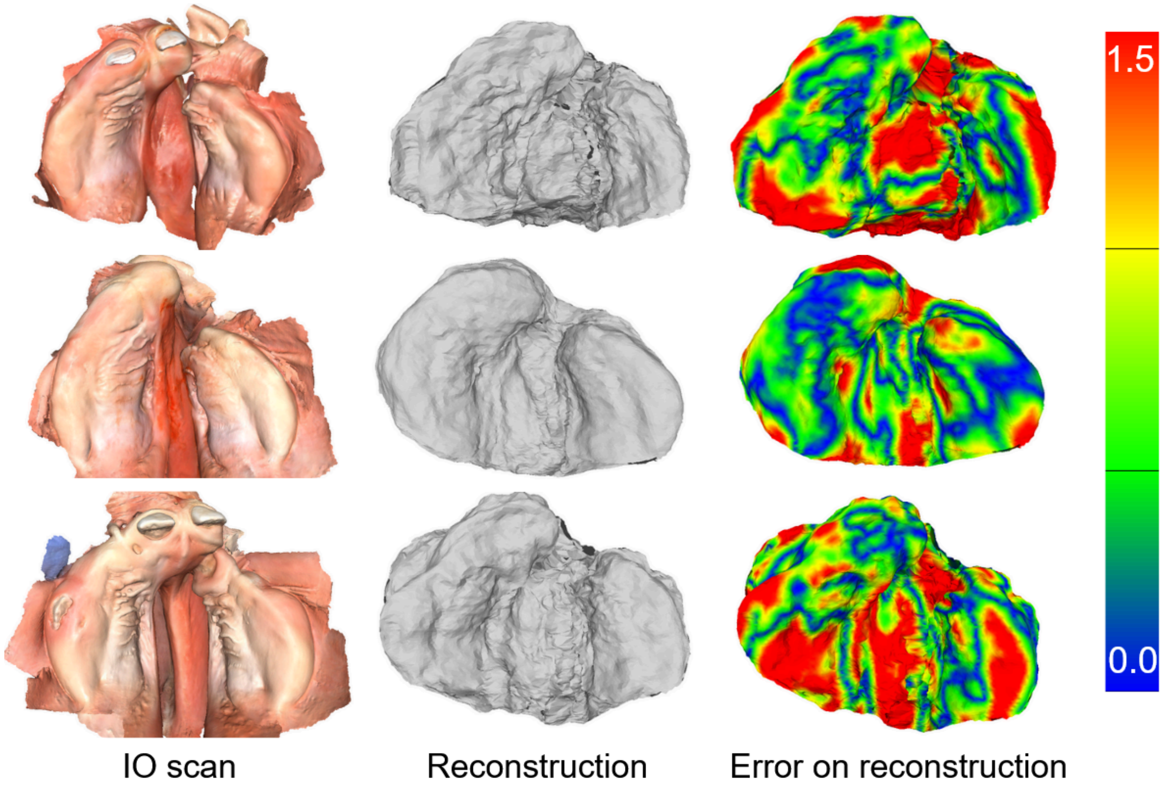

Figure 8: We present qualitative results of final reconstruction when fitting the PCA model to the predicted landmarks on real-world images. Each row represents a different identity. The first column are the captured IO scans, the middle column is the reconstruction by the PCA model fitted to predicted landmarks and the right column is the same reconstruction but with the error to the captured scan color coded. The errors are in mm. The first identity has an average error of 1.20 with a variance of 0.99, the second identity has an average error of 0.73 with a variance of 0.59 and the third identity has an average error of 1.03 with a variance of 0.77.

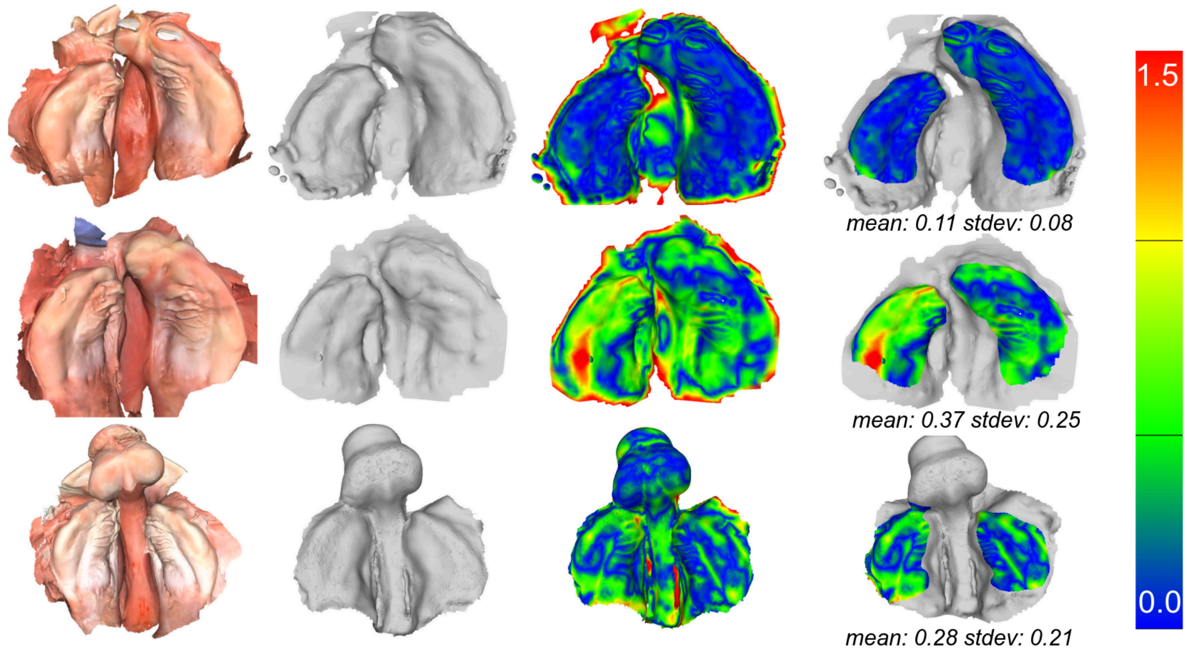

Figure 9: We show the performance of the photogrammetry approach by Lingens et al. [1] . Each row represents a different identity. The first column is the original IO scan. The second column is the reconstruction. The third has the error between reconstruction and IO scan color coded and the fourth column focuses on the error only in the region of interest. The error values given correspond only to that region.

Uncertainty Prediction:

The NGLL-loss applied during training predicts position and confidence of the landmarks. Wood et al. [3] state that the landmark predictor can learn the difference between visible and hidden landmarks during training. It assigns a higher confidence to the visible ones and a lower confidence to the hidden. When fitting the shape model to the landmarks the confidence can serve as a weight for each corresponding point when optimizing the model parameters. *Figure 10* compares the reconstruction accuracy of the PCA fitting process with and without confidence. The average error without confidence is 1.08mm and with confidence it is 1.02mm. The average variance without confidence is 0.89mm and with confidence it is 0.85mm . The PCA reconstruction is therefore more precise when considering the uncertainty predicted along the position of the points. To incorporate the confidence we optimize the rigid transformation and PCA model parameters iteratively to fit the predicted landmarks. We assign the individual loss of each point a weight inversely scaling with the variance. The smaller the variance, the higher the confidence, the more important the point is during optimization. The larger the variance, the lesser the confidence and the point weight is small.

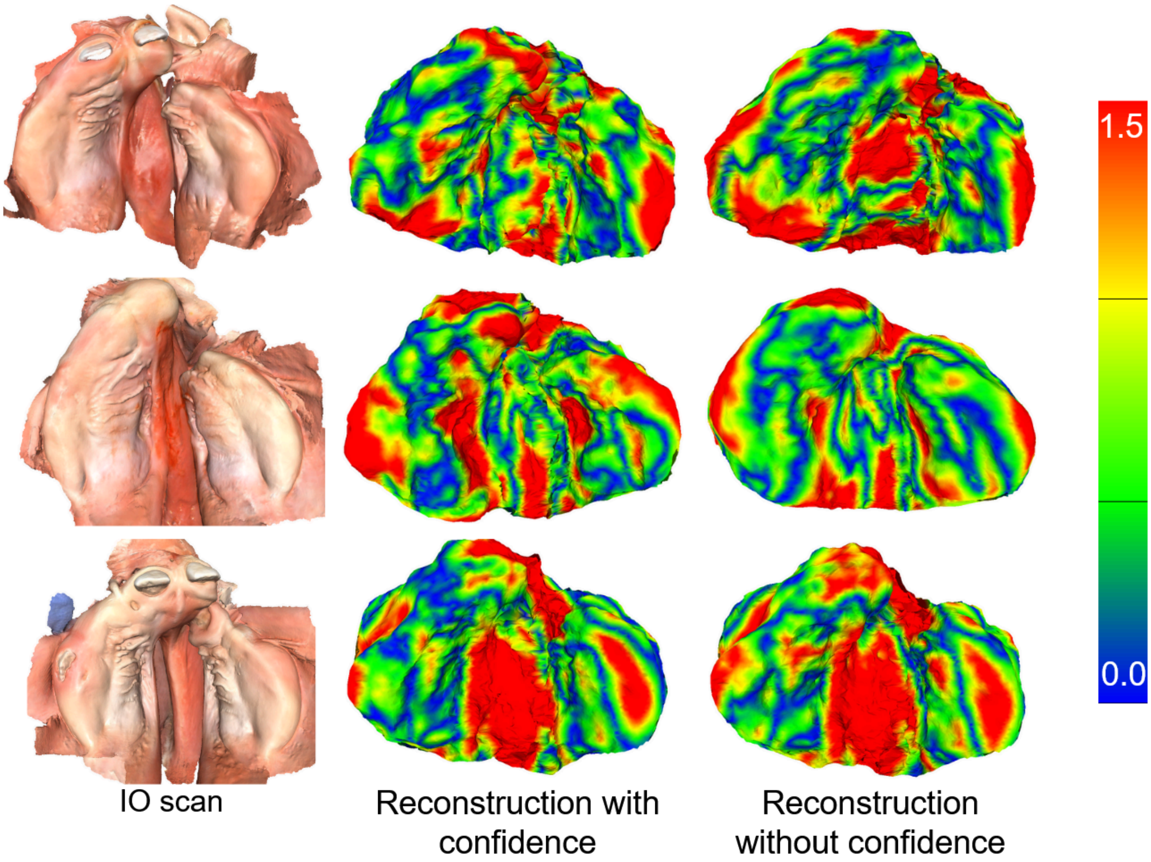

Figure 10: We present qualitative results of the PCA reconstruction when considering and when ignoring the confidence predicted by the network. Each row is a different identity. We show from left to right the captured IO scan, the reconstruction with confidence and the reconstruction without confidence. The errors are in mm.

Reprojection for Real Dataset:
We would like to thank reviewer 3 for the comments on the creation of the real dataset. They argued that instead of reprojecting the dense landmarks from the registered mesh, one should instead first project the points of the registered mesh onto the original IO scan and then reproject those into the image. The resulting points would represent the shape better. In future work, we see this as an interesting avenue for further exploration and potential improvement of our method.
Further, we present the difference of the registered meshes to the original IO scan in *Figure 11* to provide an estimate of the possible improvement. The measured distance is small compared to the errors introduced by the PCA reconstruction and landmark fitting. Note that additionally most of the errors occur in less relevant regions, such as the inner cleft. The median error of registered meshes is 0.06mm, the average 0.08 mm and the average variance is 0.29 mm. The registered meshes are utilized to build the PCA model and to reproject dense landmarks into real-world images.

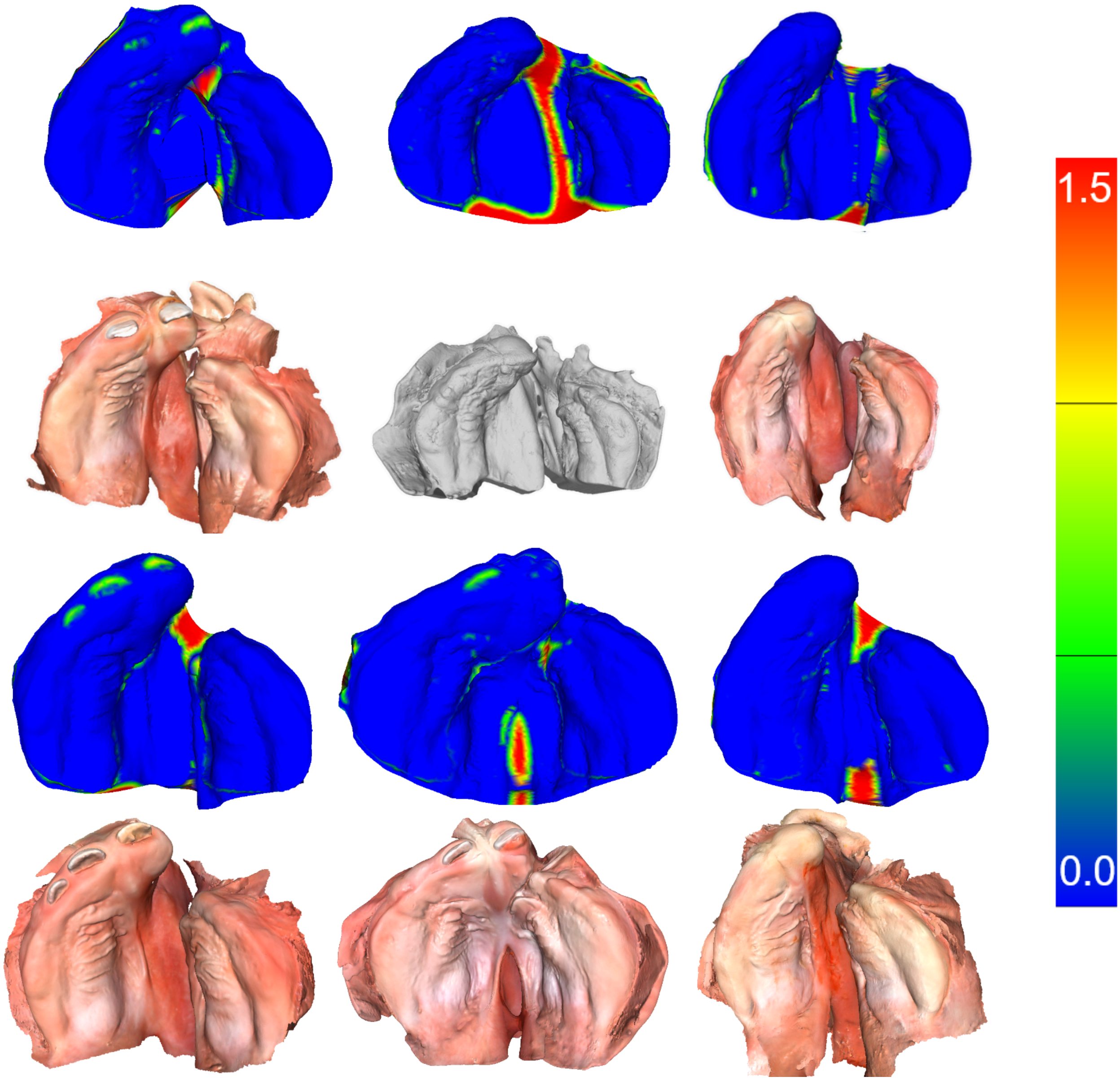

Figure 11: We show qualitative examples of the registration process. In row 2 and 4 we show the original IO scans. Above each scan is the corresponding registered mesh. The errors are in mm.

Qualitative results and failure cases:
We present qualitative results of predicted landmarks on synthetic images (*Figure 12*) and real-world images (*Figure 13*). We further present failed landmark predictions (*Figure 14*).

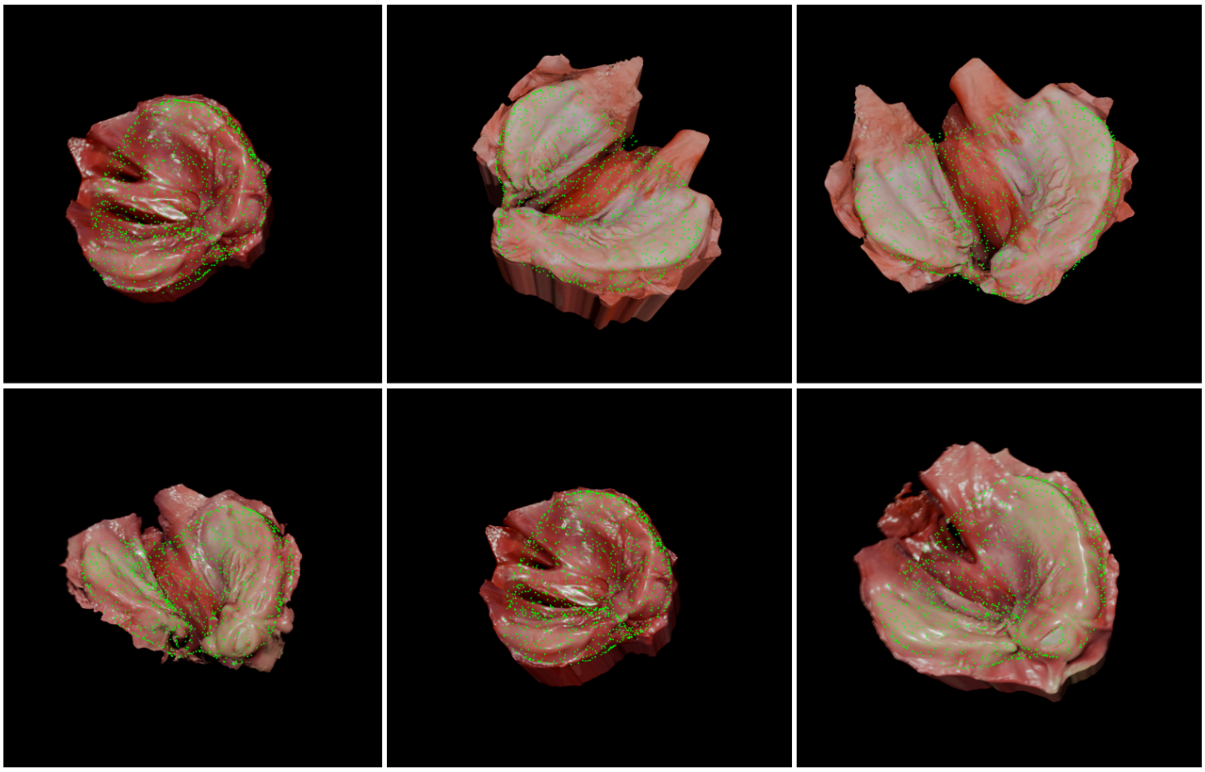

Figure 12: We present qualitative results of landmark predictions on our synthetic validation set.

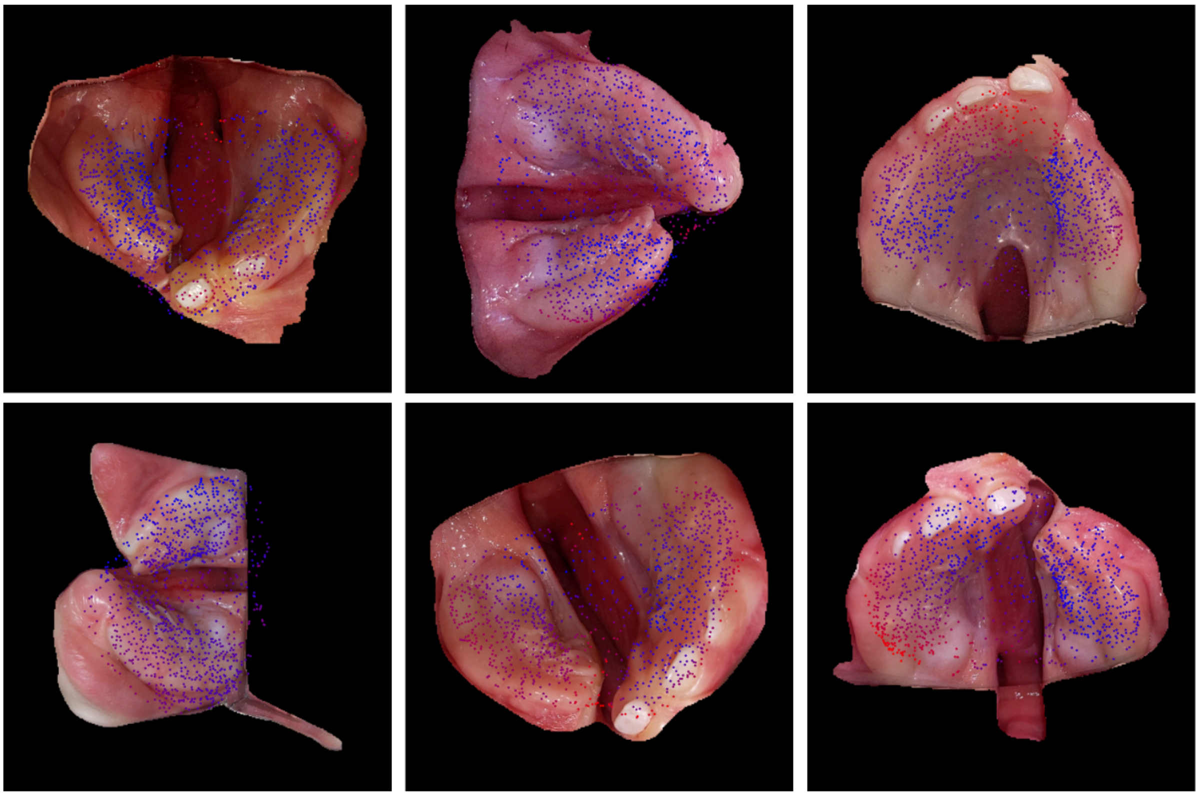

Figure 13: We present qualitative results of landmark predictions on our real-world image validation set.

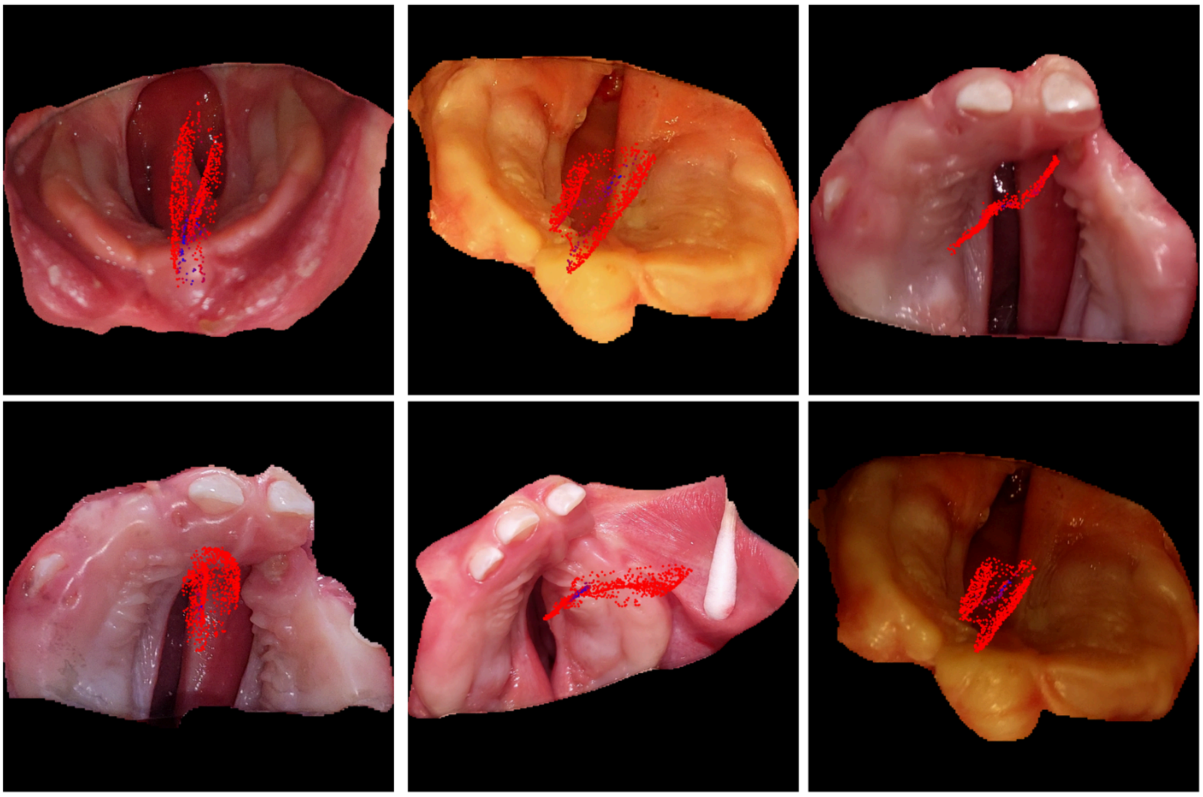

Figure 14: We present a number of failure cases of landmark predictions on our real-world image validation set.

References:

[1] Lingens, L., G¨ozc¨u, B., Schnabel, T., Lill, Y., Benitez, B.K., Nalabothu, P., Mueller, A.A., Gross, M., Solenthaler, B.: Image-based 3d reconstruction of cleft lip and palate using a learned shape prior. In: Applications of Medical Artificial Intelligence: Second International Workshop, AMAI 2023, Held in Conjunction with MICCAI 2023, Vancouver, BC, Canada, October 8, 2023, Proceedings, pp. 94–103. Springer, Berlin, Heidelberg (2023). https://doi.org/10.

[2] Schnabel, T.N., G¨ozc¨u, B., Gotardo, P., Lingens, L., Dorda, D., Vetterli, F., Emhemmed, A., Nalabothu, P., Lill, Y., Benitez, B.K., Mueller, A.A., Gross, M., Solenthaler, B.: Automated and data-driven plate computation for presurgical cleft lip and palate treatment. International Journal of Computer Assisted Radiology and Surgery (2023) https://doi.org/10.1007/s11548-023-02858-6

[3] Wood, E., Baltruˇsaitis, T., Hewitt, C., Johnson, M., Shen, J., Milosavljevi´c, N., Wilde, D., Garbin, S., Sharp, T., Stojiljkovi´c, I., et al.: 3d face reconstruction with dense landmarks. In: European Conference on Computer Vision, pp. 160–177 (2022). Springer
